# Myocardial Dysfunction after Severe Food Restriction Is Linked to Changes in the Calcium-Handling Properties in Rats

**DOI:** 10.3390/nu11091985

**Published:** 2019-08-22

**Authors:** Adriana Fernandes de Deus, Vítor Loureiro da Silva, Sérgio Luiz Borges de Souza, Gustavo Augusto Ferreira Mota, Paula Grippa Sant’Ana, Danielle Fernandes Vileigas, Ana Paula Lima-Leopoldo, André Soares Leopoldo, Dijon Henrique Salomé de Campos, Loreta Casquel de Tomasi, Carlos Roberto Padovani, Stephen C. Kolwicz, Antonio Carlos Cicogna

**Affiliations:** 1 Department of Internal Medicine, Botucatu Medical School, São Paulo State University, Botucatu 18618687, Brazil; 2 Department of Sports, Center of Physical Education and Sports, Federal University of Espírito Santo, Vitória 29075-910, Brazil; 3 Department of Biostatistics, Institute of Biosciences, São Paulo State University, Botucatu 18618970, Brazil; 4 Department of Health and Exercise Physiology, Ursinus College, Collegeville, PA 19426, USA

**Keywords:** malnutrition, heart impairment, papillary muscle assay, calcium transient proteins, SERCA2a, L-type calcium channel

## Abstract

Severe food restriction (FR) impairs cardiac performance, although the causative mechanisms remain elusive. Since proteins associated with calcium handling may contribute to cardiac dysfunction, this study aimed to evaluate whether severe FR results in alterations in the expression and activity of Ca^2+^-handling proteins that contribute to impaired myocardial performance. Male 60-day-old Wistar–Kyoto rats were fed a control or restricted diet (50% reduction in the food consumed by the control group) for 90 days. Body weight, body fat pads, adiposity index, as well as the weights of the soleus muscle and lung, were obtained. Cardiac remodeling was assessed by morphological measures. The myocardial contractile performance was analyzed in isolated papillary muscles during the administration of extracellular Ca^2+^ and in the absence or presence of a sarcoplasmic reticulum Ca^2+^-ATPase (SERCA2a) specific blocker. The expression of Ca^2+^-handling regulatory proteins was analyzed via Western Blot. Severe FR resulted in a 50% decrease in body weight and adiposity measures. Cardiac morphometry was substantially altered, as heart weights were nearly twofold lower in FR rats. Papillary muscles isolated from FR hearts displayed mechanical dysfunction, including decreased developed tension and reduced contractility and relaxation. The administration of a SERCA2a blocker led to further decrements in contractile function in FR hearts, suggesting impaired SERCA2a activity. Moreover, the FR rats presented a lower expression of L-type Ca^2+^ channels. Therefore, myocardial dysfunction induced by severe food restriction is associated with changes in the calcium-handling properties in rats.

## 1. Introduction

Previous literature demonstrated that food restriction (FR) from 10% to 40% increases longevity and prevents aging-related diseases such as diabetes mellitus, hypertension, and cancer [1,2,3,4,5]. It is suggested that an excessive availability of macronutrients, rather than a specific nutrient, results in increased oxidative stress [6]. Therefore, a reduction of total caloric intake via FR may result in beneficial adaptations that promote overall health. However, the FR associated to malnutrition in humans has detrimental health effects, particularly on cardiac function [7,8,9,10]. In experimental models, our previous studies [11,12,13,14,15,16,17,18,19,20] and studies from other laboratories [21,22,23,24] suggest that severe dietary restriction, around 50%, leads to malnutrition and depresses cardiac performance. In young and mature rats subjected to long-term severe FR, myocardial dysfunction was associated with mitochondrial failure [25], changes in ultrastructure, including myofibril density reduction and myofilament/Z line disorganization [17,20,26,27], as well as collagen accumulation [27,28] and β-adrenergic system changes [28]. Despite these findings, the intrinsic factors responsible for the impairment of cardiac function have not been elucidated.

Calcium handling is an essential process that facilitates myocardial contraction and relaxation. Previous work examined whether alterations in Ca^2+^ handling participates in the decline of myocardial function in response to malnutrition, but the results are divergent [12,13,14,15,16,17,29,30]. While one study suggested that cardiac ultrastructural changes were responsible for deleterious cardiac outcomes [17], several studies demonstrated that abnormal activity of Ca^2+^ handling specific regulatory proteins has a fundamental role in the negative repercussions of severe FR on myocardial function [13,15,16,19]. Although malnutrition was shown to alter the gene transcription process that forms proteins involved in Ca^2+^ handling [14,15,19,31], limited studies evaluated changes at the protein level. Work by De Tomasi et al. [15] focused on the L-type calcium channel, and found only a significant reduction in its protein expression in the hearts of FR rats. However, as it is known that the levels of transcripts and proteins are not always in direct correlation [32,33] and no study evaluated all the proteins involved in Ca^2+^ handling, additional work is required to understand the role that this mechanism plays in the development of cardiac dysfunction during long-term FR.

Thus, the aim of this study is to test the hypothesis that malnutrition induced by restricting the food intake causes the deterioration of myocardial function due to alterations in the expression of calcium-handling proteins and sarcoplasmic reticulum Ca^2+^-ATPase (SERCA2a) activity. The results of our study demonstrate that 90 days of FR severely diminishes cardiac function in rats, which is in part due to decreased intracellular calcium influx through the L-type channel and depressed SERCA2a activity.

## 2. Materials and Methods

### 2.1. Animal Model and Experimental Protocol

Sixty-day-old male Wistar–Kyoto rats were obtained from UNICAMP—the State University of Campinas, Brazil. The animals were randomized into control (C) and food restriction (FR) groups. C animals (n = 14) received commercial chow (3.76% fat, 20.96% protein, 52.28% carbohydrate, 9.60% fiber, and 13.40% moisture) and water ad libitum. The FR group (n = 13) was subjected to a severe food restriction equivalent to 50% of the average amount consumed by group C. Food intake for the control group was measured daily and used to calculate food quantity for the FR group. Rats were maintained on this dietary regimen for 90 days and were weighed once a week. The rats were kept in individual cages at a 23 °C room temperature, with light cycles of 12 h and relative humidity of 60%. At the end of experimental protocol, the animals were anesthetized with an intramuscular injection of ketamine (50 mg/kg; Dopalen^®^, Sespo Indústria e Comércio Ltd.a—Divisão Vetbrands, Jacareí, São Paulo, Brasil) and xylazine (10 mg/kg; Dopalen^®^, Sespo Indústria e Comércio Ltd.a—Divisão Vetbrands, Jacareí, São Paulo, Brasil), decapitated, and thoracotomized.

The project was approved by the “Committee for Experimental Research Ethics of the Faculty of Medicine in Botucatu—UNESP” (CEUA 755-2009), in accordance with the “Guide for the Care and Use of Laboratory Animals”.

### 2.2. General Characteristics

Initial (IBW) and final body weights (FBW), total body fat (BF), adiposity index (AI), naso-anal and tibia length, and soleus muscle and lung weight were measured to assess the effects of food restriction on body parameters of rats. The adipose tissue fat pads (epididymal, retroperitoneal, and visceral) were dissected and weighed. Adiposity index (AI) was calculated using the formula: AI = [total body fat (BF)/FBW] × 100. BF was measured from the sum of the individual fat pad weights as follows: BF = epididymal fat + retroperitoneal fat + visceral fat.

### 2.3. Cardiac Morphological Post Mortem Study

Cardiac remodeling was determined by macroscopic analysis of the following parameters: total weight of the heart (HW) and left ventricle (LVW), right ventricle (RVW), and atria (ATW), and normalized to body weight.

### 2.4. Myocardial Function

The cardiac contractile performance was evaluated by studying isolated papillary muscles from the LV, as previously described [34,35]. The following mechanical parameters were measured during isometric contraction: developed tension (DT; g/mm^2^), positive tension derivative (+dT/dt; g/mm^2^/s) and negative tension derivative (−dT/dt; g/mm^2^/s). The mechanical behavior of papillary muscles was assessed by increasing extracellular Ca^2+^ concentrations (0.5 mM, 1.0 mM, 1.5 mM, 2.0 mM, and 2.5 mM) in the presence and absence of cyclopiazonic acid (CPA, 30 mM), which is a highly specific blocker of SERCA2a. All the variables were normalized per cross-sectional area of papillary muscle (CSA). To avoid the central core hypoxia and impaired functional performance, papillary muscles with CSA >1.5 mm^2^ were excluded from analysis.

### 2.5. Expression of Calcium-Handling Protein

Protein expression was analyzed by Western blot. Fragments of the LV were frozen in liquid nitrogen and stored in a freezer at −80 °C. Frozen samples were homogenized with a Polytron apparatus (IKA T25 Basic Ultra Turrax TM, Wilmington, NC, USA) in hypotonic lysis buffer (50 mM of potassium phosphate pH 7.0, 0.3 M of sucrose, 0.5 mM of dithiothreitol [DTT], 1 mM of ethylenediamine tetraacetic acid [EDTA] buffer pH 8.0, 0.3 mM phenylmethylsulfonyl fluoride [PMSF], 10 mM of sodium fluoride [NaF], and phosphatase inhibitor). The homogenization product was centrifuged (5804R Eppendorf, Hamburg, Germany) at 12.000 rpm for 20 min at 4 °C, and the supernatant was transferred to tubes and stored in a freezer at −80 °C tubes. Protein concentration was analyzed by the method of Bradford [36] using the curves of Bovine Serum Albumin (BSA) Protein Standard (Bio-Rad, Hercules, CA, USA). Samples were diluted in Laemmli buffer (240 mM of Tris-HCL, 0.8% sodium dodecyl sulfate [SDS], 40% glycerol, 0.02% bromophenol blue, and 200 mM of β-mercaptoethanol) and separated via electrophoresis using Mini-Protean 3 Electrophoresis Cell (Bio-Rad, Hercules, CA, USA). Electrophoresis was performed with a biphasic gel; stacking (240 mM Tris-HCl pH 6.8, 30% polyacrylamide, 10% ammonium persulfate [APS], and tetramethylethylenediamine [TEMED]) and resolution (240 mM of Tris-HCl pH 8.8, 30% polyacrylamide, 10% APS, and TEMED) at a concentration of 6% to 10%, depending on the molecular weight of the analyzed protein. The Kaleidoscope Prestained Protein Standard (Bio-Rad, Hercules, CA, USA) was used to identify the size of the bands. Electrophoresis was performed at 120 V (Power Pac HC 3.0A, Bio-Rad, Hercules, CA, USA), for 3 h with running buffer (0.25 M of Tris, 192 mM of glycine, and 1% SDS). Proteins were transferred to a nitrocellulose membrane using a Mini Trans-Blot (Bio-Rad, Hercules, CA, USA) system with transfer buffer (25 mM of Tris, 192 mM of glycine, 20% methanol, and 0.1% SDS). Membranes were washed twice with TBS-T buffer (20 mM of Tris-HCl pH 7.4, 137 mM of NaCl, and 0.1% of Tween 20). Membranes were blocked with 5% nonfat dry milk in TBS-T for 120 min at room temperature under constant agitation. The membrane was washed three times with TBS-T and incubated with primary antibody diluted in blocking solution under constant agitation for 12 h. After incubation with the primary antibody, membranes were washed three times with TBS-T and incubated with the secondary antibody in blocking solution for 2 h under constant stirring. Then, the membrane was washed three times with TBS-T to remove secondary antibody excess. Immunodetection was performed using chemiluminescence according to the manufacturer’s instructions (Amersham Biosciences, Piscataway, NJ, USA) and detected by autoradiography. Quantification analysis of blots was performed using Scion Image software (Scion Corporation, Frederick, Maryland, EUA). Antibodies used were: Ryanodine (1:1000; ABR, Affinity BioReagents, Golden, CO, USA); Calsequestrin (1:2000; ABR, Affinity BioReagents); Exchanger Na^+^/Ca^2+^ (1:2000; Upstate, Lake Placid, NY, USA); SERCA2a (1:2500; ABR, Affinity BioReagents); Phospholamban (1:5000; ABR, Affinity BioReagents); Phospho-Phospholamban (Ser16) (1:5000; Badrilla, Leeds, West Yorkshire, UK); Phospho-Phospholamban (Thr17) (1:5000; Badrilla); L-type calcium channel (anti-α1C) (1:1000; Santa Cruz Biotechonology Inc., Santa Cruz, CA, USA); and β-Actin (1:1000; Santa Cruz Biotechnology Inc.). Secondary antibodies that were linked to peroxidase were used (IgG anti-mouse or IgG anti-rabbit, depending on the protein; 1:5000-1:10,000; Santa Cruz Biotechnology Inc.).

### 2.6. Statistical Analysis

All the data were tested for normality before statistical analysis using the Shapiro–Wilk test. Data from physical characteristics, cardiac post-mortem morphology, and Western blot of calcium-handling proteins were reported by descriptive measures of position and variability and subjected to the “t” test for independent samples. For isolated papillary muscle experiments, the adjustment of the DT, +dT/dt, and −dT/dt response linear model, in the presence and absence of CPA as a function of the extracellular Ca^2+^ concentration elevation, expressed by *Response Variable = a + b/[Ca^2+^]_extracellular_*, was performed by the minimum squares technique complemented with the comparative test of average profiles of responses regarding the parallelism and coincidence of the adjusted models to the group. The statistical analyses were performed using SigmaPlot 12.0 (Systat Software, Inc., San Jose, CA, USA). The level of significance for all variables was *p* < 0.05.

## 3. Results

### 3.1. Physical Characteristics

Severe food restriction (FR) resulted in substantial changes to the physical phenotype of the rats. As shown in Table 1, 90 days of FR significantly reduced body weight and fat mass measures, resulting in an approximate 12-fold decrease in the adiposity index. Furthermore, masses of the soleus muscle, lung, and tibia were significantly lower in FR rats. These results show that 90 days of FR severely alters physical phenotype, including the impairment and/or regression of organ growth.

### 3.2. Macroscopic Cardiac Morphology

As shown in Table 2, the weight of the left ventricle (LV), right ventricle (RV), and atria (AT) were approximately 50% lower in FR rats compared to their controls. However, the data normalized by FBW were similar between the groups.

### 3.3. Assessment of Papillary Muscle Function

**Figure 1:**
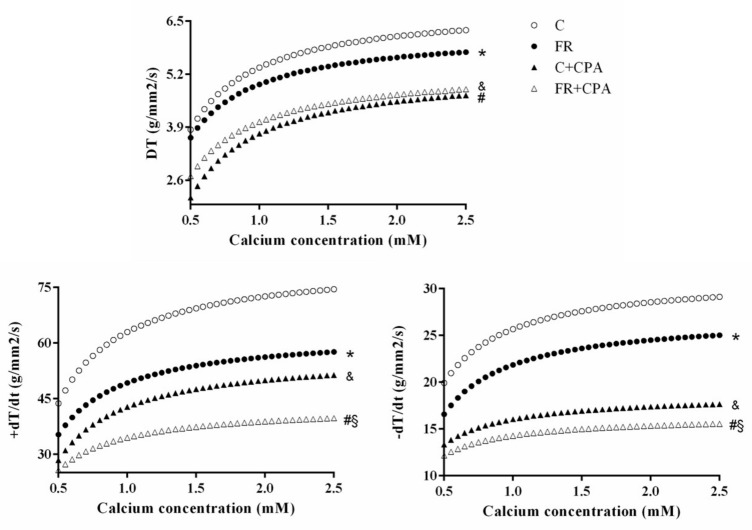
Food restriction (FR) animals presented altered functional responses in the papillary muscle assay in the following parameters: (**A**) developed tension (DT), (**B**) positive tension derivative (+dT/dt), and (**C**) negative tension derivative (−dT/dt) C: control group. CPA: Cyclopiazonic acid. C + CPA: control submitted to CPA; FR + CPA: food restriction submitted to CPA. Data presented as mean ± standard deviation. Minimum squares technique complemented with the comparative test of average profiles of responses. *p* < 0.05; * C vs. FR; § C + CPA vs. FR + CPA; # FR vs. FR + CPA; and & C vs. C + CPA.

**Figure 2:**
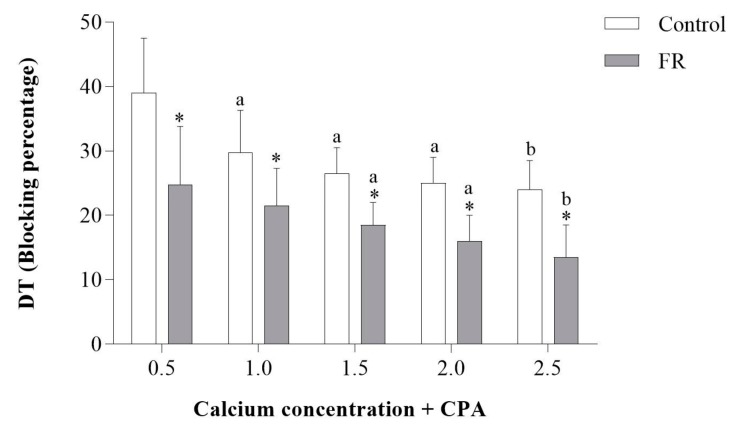
Blocking percentage under cyclopiazonic acid in papillary muscle preparation. C: control; FR: food restriction; CPA: cyclopiazonic acid; DT: developed tension. Data are expressed as mean ± standard deviation. Repeated measures two-way ANOVA complemented with Bonferroni post-hoc test. *p* < 0.05. ^a^ vs. 0.5 mM Ca^2+^ concentration; ^b^ vs. 0.5 and 1.0 mM Ca^2+^ concentration; * vs. control. (*n* = 13–14 each group).

To determine whether abnormal heart size was associated with myocardial dysfunction, the contractile performance of LV papillary muscles isolated from control and FR hearts was measured. In response to increasing extracellular Ca^2+^ concentration, the developed tension (DT) was significantly reduced in papillary muscles from FR hearts (Figure 1A). Papillary muscles from FR hearts also had significantly lower values for positive tension derivative (+dT/dt) and negative tension derivative (−dT/dt) (Figure 1B,C), which is consistent with impaired contractility and relaxation. Additionally, the administration of cyclopiazonic acid (CPA), which is a specific inhibitor of SERCA2a, further decreased +dT/dt and −dT/dt (Figure 1B,C) in FR hearts. Moreover, Figure 2 shows that the blocking percentage by CPA was lower in the FR than the control group. This result suggests a prior impairment of SERCA2a activity under caloric restriction, which would reduce the magnitude of the effect of cyclopiazonic acid inhibition. Overall, these findings show that 90 days of FR severely impairs myocardial contraction and relaxation, which is likely due to depressed SERCA2a activity.

### 3.4. Expression of Calcium-Handling Protein

**Figure 3:**
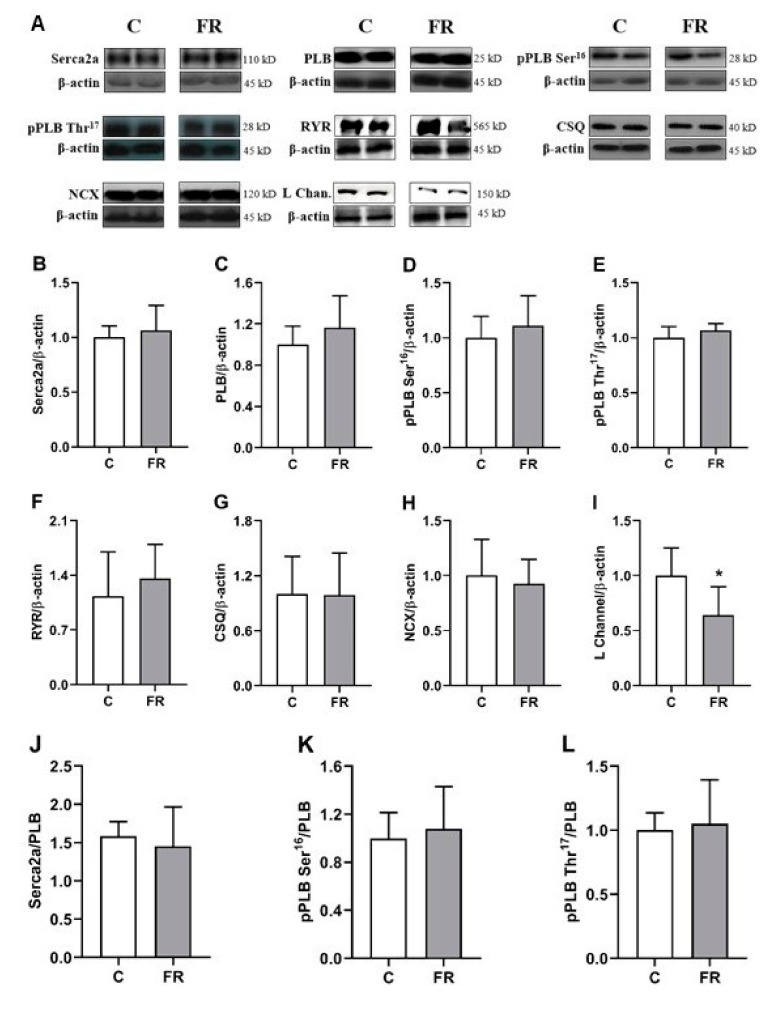
Protein expression of calcium-handling regulators evaluated by Western blot in the myocardium from control (C) and food restriction groups (FR) (n = 6 in each group). (**A**) Representative bands of the proteins. Quantification of myocardial (**B**) sarcoplasmic reticulum calcium-ATPase (SERCA2a), (**C**) total phospholamban (PLB), (**D**) phosphorylated PLB on serine-16 (pPLB Ser^16^), (**E**) phosphorylated PLB on threonine-17 (pPLB Thr^17^), (**F**) ryanodine receptor (RYR), (**G**) calsequestrin (CSQ), (**H**) sodium-calcium exchanger (NCX), and (**I**) L-type Ca^2+^ channel (L Channel) normalized to β-actin (internal control). Quantification of (**J**) SERCA2a, (**K**) pPLB Ser^16^, and (**L**) pPLB Thr^17^ normalized to total PLB. Data are expressed as mean ± standard deviation. Student’s *t*-test for independent samples. * *p* < 0.05 vs. C.

Calcium-handling proteins are integral in facilitating myocardial contraction and relaxation. Therefore, a Western blot analysis was conducted focusing on the primary proteins involved in calcium handling, including SERCA2a, phospholamban (PLB), ryanodine receptor (RYR), calsequestrin (CSQ), sodium-calcium exchanger (NCX), and the L-type calcium channel (L channel). As shown in Figure 3B,F–H, no statistical differences were noted in SERCA2a, RYR, CSQ, or NCX. Additionally, no differences in PLB or the phosphorylation sites at PLB Ser^16^ or PLB Thr^17^ were observed (Figure 3C–E). However, 90 days of FR did result in a significant lowering of L-channel protein levels (Figure 3I). Furthermore, the ratios of SERCA2a/PLB, phosphorylated PLB (pPLB) Ser16/PLB, pPLB Thr17/PLB were similar in both groups (Figure 3J–L).

## 4. Discussion

The purpose of this study was to investigate the contribution of alterations in the expression of Ca^2+^-handling proteins and SERCA2a activity to myocardial performance during severe food restriction. The nutritional protocol used in this study was sufficient in duration and intensity to cause malnutrition, which is in agreement with previous studies [12,13,17]. The main findings are that malnutrition reduces the myocardial L-type calcium channels protein expression and alters SERCA2a activity, as expressed by the decrement of the mechanical responses to extracellular Ca^2+^ elevation in the absence and presence of cyclopiazonic acid. Overall, these changes in the calcium-handling capacity of the cardiac myocyte are critical in the development of myocardial dysfunction during severe food restriction.

In agreement with previous literature [11,12,13,14,15,17,19,31], the 50% reduction of food intake used in this study promoted drastic changes in the body composition of animals, as visualized by a reduction in all of the variables related to fat and body weight. Malnutrition causes damage to several segments of the body system, including a substantial reduction of hind limb muscles such as the soleus, plantaris, gastrocnemius, and extensor digitorum longus muscles [12,13,15,37,38]. In addition, food restriction leads to severe changes in the cardiorespiratory structures, particularly the weights of the lungs, atria, and ventricles [13,14,16]. These above responses, which were also observed in our study, are consistent with increased protein catabolism, as well as decreased protein synthesis, since the restriction of pellet intake produces protein and energy deficiency [38].

In isolated papillary muscle analysis, malnutrition resulted in decreased contractile (DT and +dT/dt) and relaxation (−dT/dt) capacity, which is in accordance with functional impairments observed in precedent studies [12,13,19,21,22,24,39]. At the molecular level, the quantity and quality of contractile proteins and the availability of energy and Ca^2+^ are crucial factors in the regulation of myocardial performance [40,41]. Previous work from our laboratory showed that severe food restriction causes a reduction of myofibril density and myofilament disorganization [12,17,20,26]. Furthermore, the authors reported myosin isoform remodeling with the down-regulation of alpha- (V_1_) and up-regulation of beta-myosin heavy chain (V_3_) genes, which present faster and lower ATPase activity, respectively [24,30]. In addition, the constant low availability of substrates during the malnutrition period may lead to mitochondrial injury [17,20], leading to a lack of ATP production, which may impair the capacity of the ATP-dependent proteins.

The primary aim of this study was to evaluate the role of Ca^2+^-handling proteins in the development of myocardial dysfunction induced by malnutrition. Myocardial Ca^2+^-handling inadequacies are among the most critical responses to cardiac aggression [12,13,14,15,16,17,19,29,30,31]. Previous studies reported alterations in the activity [13,15,16] and content [14,15,31] of Ca^2+^-handling regulatory components, which is consistent with our current observations. With any cardiac remodeling process at the macro or micro level, the initial intent of adaptations in Ca^2+^ handling is to attenuate the insult imposed on the myocardium in an effort to maintain the functional balance. However, these adaptations are incapable of enduring the aggression over time, contributing to the establishment of the dysfunctional condition [42,43]. In the present study, 90 days of severe food restriction resulted in alterations in SERCA2a activity and, among the analyzed proteins, only a reduction in L-type calcium channels protein expression. During the Ca^2+^ transient, the SERCA2a is an ATP-dependent protein that is responsible for the reuptake of Ca^2+^ into the sarcoplasmic reticulum of the cardiomyocyte, which is crucial for the maintenance of the cytosolic concentration. Although we did not observe a reduction of SERCA2a protein expression, impaired activity was noted in the papillary muscle assay in the presence of cyclopiazonic acid, as noted previously [19]. This finding is likely due to the low-energy capacity in severe food restriction, which limits the ATP required for proper SERCA2a function. Impaired SERCA2a activity may provoke the elevation of diastolic Ca^2+^, which would lead to the increased importance of the exchanger Na^+^/Ca^2+^ (NCX) cytosolic Ca^2+^ extrusion. Since the NCX mechanism has a reduced capacity to remove cytosolic Ca^2+^, alterations in Ca^2+^ handling persist.

**Figure 4:**
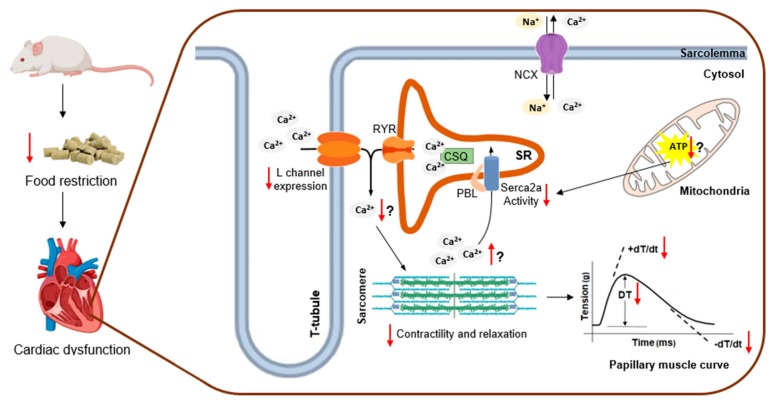
Overview of food restriction effects over myocardium and presumable interactions between molecular changes and myocardial mechanical impairment. Food restriction reduced sarcoplasmic reticulum Ca^2+^-ATPase (SERCA2a) activity and L-type calcium channel (L channel) expression and impaired myocardial mechanical performance. Hypothetical adenosine triphosphate (ATP) depletion due to malnutrition could reduce the SERCA2a activity, leading to a high amount of residual cytosolic calcium. In turn, residual calcium signals to deplete the release of calcium by the ryanodine receptor (RYR), which is achieved by the reduced calcium entry secondary to the decreased protein expression of the L channel. This sequence of events involving the myocardial calcium handling would be a plausible mechanism by which the food restriction deteriorates the cardiac function. Ca^2+^, calcium; Na^+^, sodium; NCX, sodium-calcium exchanger; SR, sarcoplasmic reticulum; CSQ, calsequestrin; PLB, phospholamban; DT, developed tension; +dT/dt, positive tension derivative; −dT/dt negative tension derivative.

In disagreement with our hypothesis, in which changes in several Ca^2+^-handling proteins were expected, our study showed only reduction in the expression of the L-type calcium channel protein due to food restriction, which is in accordance with a previous report [15]. Although the reason for this outcome is unclear, we speculate that this may be an adaptive response, serving as a cellular defense mechanism in response to dysfunctional SERCA2a activity. In this regard, myocyte Ca^2+^ influx is decreased in order to avoid Ca^2+^ accumulation, particularly during diastole. However, the reduction of Ca^2+^ influx across the plasma membrane as a result of decreased L-type calcium channel expression over time may lead to impairments in cardiomyocyte force development, as evidenced by reduced values of DT and +dT/dt in animals suffering from malnutrition. Although the functional control of the L-type channel is well described, little is known about the processes involved in the expression of this sarcolemma channel. The L-type channel is composed of three subunits (α_1C_, α_2_δ, and β) [44], with the α_1C_ pore-forming subunit analyzed in this study. The expression of the α_1C_ subunit protein is regulated by β and α adrenergic systems [45], angiotensin II [46], and calcium flux through the L-type channel [47,48,49]. Based on our results, it is possible that the cytosolic Ca^2+^ “leftover”, due to SERCA2a dysfunction, activated the auto-regulation mechanism of L-type channel protein expression on the distal carboxyl-terminus via a process sensitive to Ca^2+^ flux alterations [47,48,49]. In conditions in which there is undue cytosolic Ca^2+^ accumulation, the hyperactivation of this regulatory mechanism may be related to the decrease in the protein expression of these channels. For better visualization of the food restriction effects over the myocardium, we constructed a figure placing the main molecular alterations and suggested a hypothesis that attributed cardiac dysfunction to malnutrition, as can be seen in Figure 4.

Therefore, our results show that malnutrition, due to 90 days of severe food restriction, leads to cardiac dysfunction. The deterioration of myocardial function may be the consequence of a reduction of L-type calcium channels protein expression and impaired SERCA2a activity. Future studies are required to understand the mechanisms that cause inadequacies in L-type Ca^2+^ calcium channels and SERCA2a activity during severe food restriction, which ultimately contribute to malnutrition-induced myocardial dysfunction.

## Figures and Tables

**Table 1:** Physical characteristics of animals.

	Groups	
	C (*n* = 14)	FR (*n* = 13)
IBW (g)	313 ± 41.2	301 ± 35.2
FBW (g)	445 ± 39.1	228 ± 19.1 *
Food intake (g/day)	21.1 ± 2.2	10.6 ± 1.1
Epididymal fat (g)	9.60 ± 3.42	0.90 ± 0.56 *
Retroperitoneal fat (g)	7.00 ± 2.80	0.19 ± 0.12 *
Visceral fat (g)	5.24 ± 1.68	0.67 ± 0.33 *
Total body fat (g)	21.8 ± 6.90	1.75 ± 0.70 *
Adiposity index	4.86 ± 1.45	0.76 ± 0.27 *
Naso-anal length (cm)	27.5 ± 0.70	24.6 ± 0.80 *
Soleus muscle (g)	0.19 ± 0.03	0.10 ± 0.01 *
Lung (g)	2.00 ± 0.41	1.19 ± 0.11 *
Tibia length (cm)	4.38 ± 0.07	4.15 ± 0.03 *
IBW/FBW ratio (g/g)	0.71 ± 0.10	1.33 ± 0.19 *

Data expressed as mean ± standard deviation. C: control group; FR: restriction food group; IBW: initial body weight; FBW: Final body weight. Student “t” test for independent samples. * *p* < 0.05 vs. C.

**Table 2:** Cardiac morphological post-mortem data.

	Groups	
	C (*n* = 14)	FR (*n* = 1 3)
LV (g)	0.84 ± 0.10	0.44 ± 0.05 *
RV(g)	0.25 ± 0.04	0.12 ± 0.01 *
AT (g)	0.10 ± 0.02	0.05 ± 0.01 *
Total heart(g)	1.20 ± 0.15	0.62 ± 0.07 *
LV/FBW (mg/g)	1.90 ± 0.22	1.93 ± 0.13
RV/FBW (mg/g)	0.56 ± 0.08	0.53 ± 0.03
AT/FBW (mg/g)	0.24 ± 0.04	0.24 ± 0.02
Heart/FBW (mg/g)	2.70 ± 0.32	2.70 ± 0.16

Data expressed as mean ± standard deviation. C: control; FR: food restriction; LV: left ventricular weight, RV: right ventricle weight, ATW: atrium weight; Heart: heart weight; FBW: final body weight. Student “t” test for independent samples. * *p* < 0.05 vs. C.
